# Genetic characterization of Japanese encephalitis virus genotype II strains isolated from 1951 to 1978

**DOI:** 10.1099/vir.0.027110-0

**Published:** 2011-03

**Authors:** Amy J. Schuh, Robert B. Tesh, Alan D. T. Barrett

**Affiliations:** 1 Center for Biodefense and Emerging Infectious Diseases, University of Texas Medical Branch, Galveston, TX 77555-0609, USA; 2 Sealy Center for Vaccine Development, University of Texas Medical Branch, Galveston, TX 77555-0609, USA; 3 Institute for Human Infections and Immunity, University of Texas Medical Branch, Galveston, TX 77555-0609, USA; 4 Department of Pathology, University of Texas Medical Branch, Galveston, TX 77555-0609, USA

## Abstract

Japanese encephalitis virus (JEV), the prototype member of the JEV serocomplex, genus *Flavivirus*, family *Flaviviridae*, is the most significant arthropod-borne encephalitis worldwide in terms of morbidity and mortality. At least four genotypes (GI–GIV) of the virus have been identified; however, to date, the genomic nucleotide sequence of only one GII virus has been determined (FU strain, Australia, 1995). This study sequenced three additional GII strains of JEV isolated between 1951 and 1978 in Korea, Malaysia and Indonesia, respectively, and compared them with the FU strain, as well as with virus strains representing the other three genotypes. Based on nucleotide and amino acid composition, the genotype II strains were the most similar to GI strains; however, these two genotypes are epidemiologically distinct. Selection analyses revealed that the strains utilized in this study are under predominantly purifying selection, and evidence of positive selection was detected at aa 24 of the NS4B protein, a protein that functions as an alpha/beta interferon signalling inhibitor.

## INTRODUCTION

Japanese encephalitis virus (JEV) is a member of the JEV serocomplex within the genus *Flavivirus*, family *Flaviviridae*. Worldwide, JEV is the most important arthropod-borne viral encephalitis, with 50 000 cases and 10 000 deaths reported yearly, although this may be a significant underestimate due to a lack of surveillance and underreporting (Barrett, 2008). Whilst most human infections are asymptomatic, approximately 25 % of symptomatic infections are fatal, 50 % develop severe, often long-term, neurological and/or psychiatric sequelae, and 25 % resolve fully (Barrett, 2008). The virus is transmitted in an enzootic cycle involving waterbirds, domestic pigs and rice paddy-breeding mosquitoes, principally *Culex tritaeniorhynchus*. Humans and other non-avian vertebrates are only infected incidentally and are considered to be dead-end hosts because they fail to produce viraemias of sufficient titres to infect mosquitoes. The prototype strain of JEV was isolated in Japan in 1935 (Lewis *et al.*, 1947) and the virus has since been found throughout East and South-east Asia with the geographical borders of virus activity extending north to maritime Siberia (Grascenkov, 1964), west to Pakistan (Igarashi *et al.*, 1994) and south-east to Australia (Hanna *et al.*, 1996).

JEV virions are approximately 50 nm in diameter and contain a host-derived lipid envelope that surrounds the nucleocapsid. Enclosed within the nucleocapsid is one molecule of positive-sense ssRNA of approximately 11 kb. This molecule contains 5′ and 3′ untranslated regions (UTRs) and a single ORF that encodes a 3432 aa polyprotein, which is co- and post-translationally cleaved by viral and host proteases into three structural proteins: the capsid protein (C), the precursor (prM) of the membrane protein, and the envelope protein (E), as well as seven non-structural proteins (NS): NS1, NS2A, NS2B, NS3, NS4A, NS4B and NS5 (Chambers *et al.*, 1990). At least four genotypes of JEV have been identified based on nucleotide sequence information derived from a 240 nt region spanning a portion of the C and prM genes, as well as E gene nucleotide sequence data. Genotype I (GI) comprises strains isolated in northern Thailand, Cambodia, Korea, China, Japan, Vietnam, Taiwan and Australia between 1967 and the present; it has been the subject of numerous genetic studies due to its recent geographical expansion and displacement of GIII of the virus (Nga *et al.*, 2004; Nitatpattana *et al.*, 2008). JEV GII includes strains that have been isolated sporadically between 1951 and 1999 in Korea, southern Thailand, Malaysia, Indonesia, Papua New Guinea and northern Australia. Historically, the south-eastern limit of JEV activity was considered to lie west of the Wallace line (Williams *et al.*, 2000). However, in 1995, an outbreak due to GII of JEV and consisting of three human cases, two of which were fatal, occurred on Badu Island in Australia's Torres Strait. GIII has been the source of numerous Japanese encephalitis epidemics throughout history, and includes strains isolated in mostly temperate regions of Asia between 1935 and the present. Curiously, GIV includes strains isolated only from mosquitoes on three islands encompassing the Indonesian archipelago between 1980 and 1981 (Chen *et al.*, 1990, 1992).

The database of wild-type JEV strains for which there is ORF nucleotide sequence information consists of relatively few isolates, and surprisingly only one of these strains belongs to GII (FU strain, Australia, 1995). The absence of genomic information on other GII strains has prevented us from obtaining a firm understanding of the genetic variation and phylogenetic relationships among strains of JEV; it also has precluded an investigation to determine whether selection and codon usage bias exist among strains of the virus. Therefore, we determined the nucleotide sequence of the ORF of three additional GII strains of JEV that were isolated from specimens collected in Korea, Indonesia and Malaysia between 1951 and 1978 and compared them with sequence data derived from the GII strain that was isolated in Australia in 1995, as well as with 28 JEV strains representative of the other three genotypes, which were isolated throughout East and South-east Asia between 1935 and 2007. In addition, selection and codon usage analyses were performed on the ORF nucleotide sequence dataset.

## RESULTS

### Phylogenetic relationships among the four genotypes of JEV and among GII strains of the virus

**Fig. 1.**
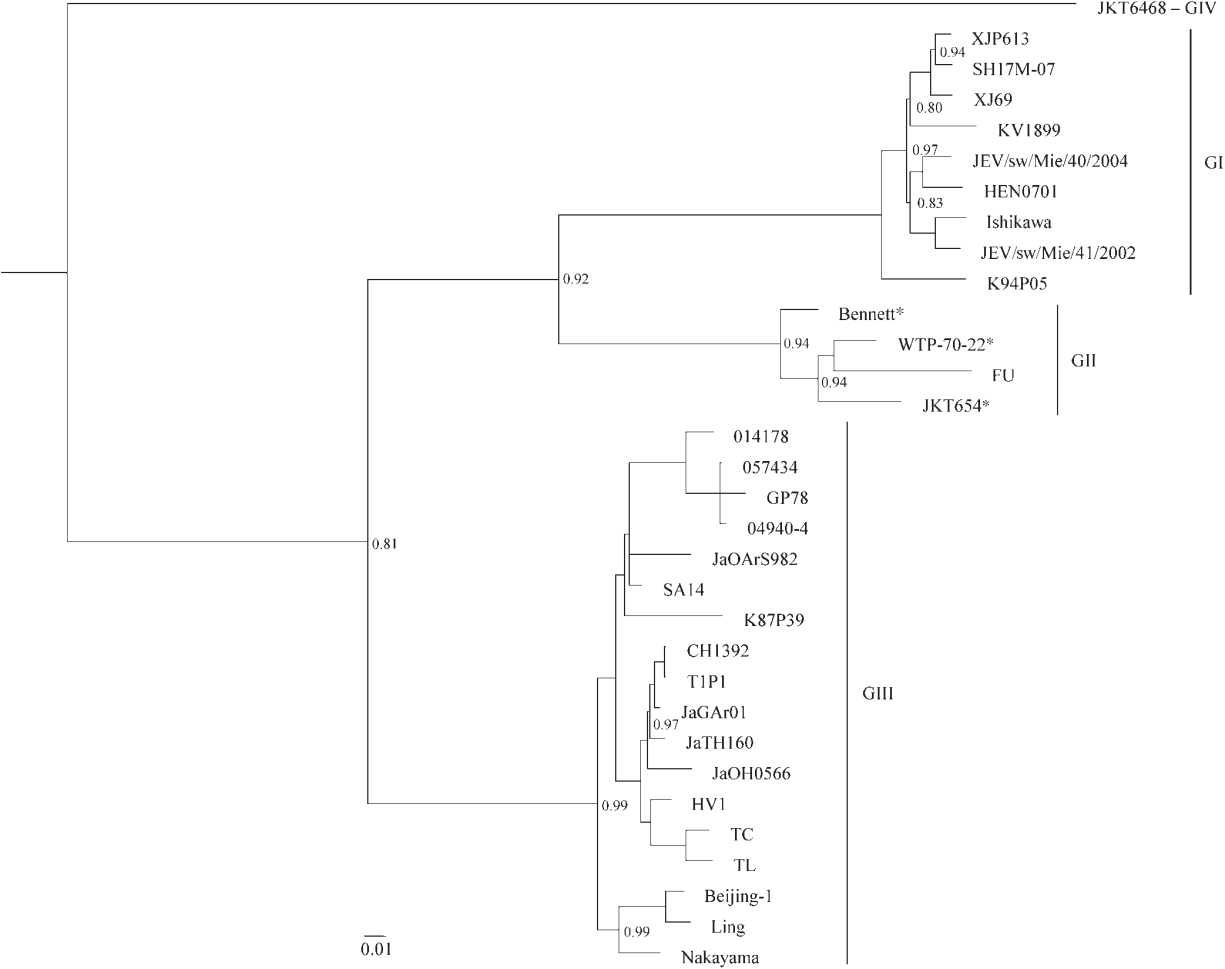
Bayesian phylogeny based on nucleotide sequence information derived from the ORF of the JEV strains. The tree was rooted with MVEV (strain MVE-1-51), which is a member of the Japanese encephalitis serogroup, but this has been omitted from the figure to allow better visualization of branch lengths. Horizontal branch lengths are proportional to the genetic distance between strains. Bar, number of nucleotide substitutions per site. GI–GIV are indicated to the right of the tree. All nodes within the phylogeny were supported by a posterior probability of 1.0 unless otherwise indicated to the right of the node. The strains sequenced in this study are indicated by asterisks.

The nucleotide sequence of the ORF of the three additional strains of GII of JEV were determined [Bennett, (Korea, *c*.1951); WTP-70-22 (Malaysia, 1970); and JKT 654 (Indonesia, 1978)]. These were compared with 29 wild-type homologous JEV sequences and one sequence of Murray Valley encephalitis virus (MVEV) that were retrieved from GenBank for a final dataset comprising 33 virus strains, each of which was 10 308 nt (3436 aa) in length (Table 1). All three methods of phylogenetic inference [Bayesian, neighbour-joining (NJ) and maximum-likelihood (ML)] confirmed previous studies and identified four major clades (representing GI–GIV), as well as revealing identical tree topologies and virus groupings (Fig. 1). Genotypes of JEV in this study were defined as strains that differed in nucleotide composition by more than 9.1 % over the entire ORF.

Nine of the 32 JEV strains belonged to GI, four strains belonged to GII (including the three strains sequenced in this study), 18 strains belonged to GIII and one strain belonged to GIV. GIV of JEV diverged from MVEV first, followed by GIII, GII and lastly GI.

Of the GII strains, the Bennett strain (Korea, *c*.1951) diverged first and was followed by divergence of the JKT654 strain (Indonesia, 1978) and finally the WTP-70-22 (Malaysia, 1970) and FU (Australia, 1995) strains, which form a monophyly.

### Genetic divergence among the four genotypes of JEV

Pairwise comparisons of both nucleotide and amino acid sequences were used to determine the genetic relatedness of GII strains to the other three genotypes of JEV (Table 2). Based on nucleotide sequence divergence, GII strains were the most similar to GI strains [9.1 % (Bennett|JEV/sw/Mie/40/2004) to 10.7 % (FU|K94P05, KV1899)], followed by GIII strains [10.2 % (Bennett|JaGAr01) to 11.7 % (FU|GP78)] and lastly the GIV strain [16.2 % (Bennett|JKT6468) to 16.6 % (FU|JKT6468)]. This same pattern held true when the amino acid sequence divergence was examined: GII strains were the most closely related to GI strains [1.5 % (Bennett|JEV/sw/Mie/40/2004, HEN0701) to 3.3 % (FU|K94P05)], followed by GIII strains [1.7 % (Bennett|JaGAr01, K87P39) to 3.0 % (FU|TC)], and finally the GIV strain [5.0 % (WTP-20-22|JKT6468) to 5.4 % (FU|JKT6468)].

A number of amino acid substitutions were specific for individual genotypes (genotype-specific amino acid substitutions), and some defined individual nodes within the phylogeny (node-defining amino acid substitutions) (see Supplementary Table S1, available in JGV Online). Genotype IV possessed 144 genotype-specific amino acid substitutions (20 in the C protein, 15 in the prM protein, 22 in the E protein, 17 in the NS1 protein, 11 in the NS2A protein, 15 in the NS3 protein, seven in the NS4A protein, eight in the NS4B protein and 29 in the NS5 protein), GIII had 17 genotype-specific substitutions (three in the C protein, two in the E protein, two in the NS1 protein, three in the NS2A protein, three in the NS3 protein, one in the NS4B protein and three in the NS5 protein), GI+GII had 27 node-defining substitutions (three in the C protein, one in the prM protein, three in the E protein, three in the NS1 protein, four in the NS2A protein, one in the NS2B protein, four in the NS3 protein, three in the NS4B protein and five in the NS5 protein), GII had 23 genotype-specific substitutions (one in the C protein, one in the prM protein, four in the NS1 protein, six in the NS2A protein, one in the NS2B protein, two in the NS3 protein, one in the NS4A protein and seven in the NS5 protein) and GI had 21 genotype-specific substitutions (one in the C protein, three in the prM protein, one in the E protein, four in the NS1 protein, one in the NS2A protein, two in the NS2B protein, three in the NS3 protein, one in the NS4A protein and five in the NS5 protein).

### Genetic diversity within GII of JEV

The intra-genotypic nucleotide and amino acid divergence were calculated to determine the extent of genetic variation within each genotype (Table 2; only the intra-genotypic divergence within GII is shown). The intra-genotypic nucleotide sequence divergence ranged from 0.7 % (XJP613|SH17M-07) to 3.2 % (K94P05|KV1899) among GI strains, from 2.5 % (Bennett|WTP-70-22) to 4.3 % (JKT654|FU) among GII strains, and from 0.1 % (CH1392|T1P1) to 4.0 % (Beijing-1|GP78) among GIII strains. The amino acid sequence divergence ranged from 0.3 % (JEV/sw/Mie/40/2004|SH17M-07 and HEN0701|JEV/sw/Mie/41/2002) to 2.8 % (K94P05|KV1899) among GI strains, 0.5 % (Bennett|WTP-70-22, Bennett|JKT654 and WTP-70-22|JKT654) to 0.9 % (JKT654|FU) among GII strains, and 0.3 % (04940-4|57434, JaGAr01|CH1392, JaGAr01|JaOArS982 and JaGAr01|JaTH160) to 2.1 % (TC|GP78) among GIII strains.

Table 3 shows the amino acid substitutions within the ORF that were specific to the four strains of GII of JEV. Compared with the Bennett strain, the WTP-70-22 strain exhibited 255 nucleotide differences resulting in 17 aa substitutions, the JKT654 strain exhibited 309 nucleotide differences resulting in 15 aa substitutions and the FU strain exhibited 402 nucleotide differences resulting in 26 aa substitutions. Interestingly, the NS2B protein was conserved among the four GII strains of JEV.

### Positive selection operating at a single site within the NS4B protein gene of JEV

Given that recombination in a nucleotide sequence alignment can adversely affect the results of selection analyses, we performed recombination analyses on the JEV sequence alignment prior to selection analyses using rdp, geneconv, Chimaera, MaxChi and Bootscan methods implemented within the rdp3 program. Preliminary analyses of the nucleotide sequences yielded ten statistically significant (*P*<0.05) potential recombination events that were detected by at least two of the recombination detection methods. However, after manual verification of the potential recombination events, using phylogenetic and recombination signal analysis features in the rdp3 program, only four of the ten potential recombination events were confirmed (see Supplementary Table S2, available in JGV Online). All confirmed recombination events identified K94P05 (Korea, 1994, mosquito, GI) as the recombinant and JaOArS982 (Japan, 1982, mosquito, GIII) as the minor parent, whilst the major parent was identified as a different GI strain in each of the four recombination events. As the K94P05 strain was not available in our collection to resequence, we were unable to determine whether the strain represented a true recombination event that occurred in nature or whether the nucleotide sequence of this strain that was submitted to GenBank represents a laboratory or sequence assembly error. Therefore, the K94P05 strain was removed from all subsequent analyses, given the fact that recombinant sequences can confound the results of evolutionary studies.

Evidence of non-neutral selection was assessed using 44 datasets that were created according to protein and genotype (Table 4). The ratio of non-synonymous (*d*_N_) to synonymous (*d*_S_) nucleotide substitutions over the first 9999 nt of the ORF for all strains was estimated to be 0.035, which suggested predominantly purifying selection. Estimates for separate genes for all strains ranged from 0.014 for the NS2B protein to 0.147 for the C protein. When the ORF was examined, estimates of the *d*_N_/*d*_S_ ratio ranged from 0.020 for GII to 0.067 for GI. This pattern persisted when the ORF dataset was parsed into individual proteins: GII consistently had the lowest *d*_N_/*d*_S_ ratio when compared with the other genotypes in seven of the ten individual genes examined, whilst GI and GIII consistently had higher *d*_N_/*d*_S_ ratios when compared with GII in all of the individual genes examined. These data suggested that GI and GIII viruses are under stronger purifying selection compared with GII viruses.

The maximum number of codons that exhibited patterns of negative selection when the all ORF strains were examined was 1432, using the random effects likelihood (REL) method, and the maximum number of codons that exhibited patterns of positive selection was ten using the REL method. However, only one codon, at site 2296 of the ORF (aa 24 of the NS4B protein), was identified as being under strong positive selection using three of the four selection detection methods. When the selection analysis was performed according to gene for all of the strains, aa 24 of the NS4B protein was again identified as being under strong positive selection using two of the four selection detection methods: fixed effects likelihood (FEL) (*P*=0.03) and internal FEL (IFEL) (*P*=0.04).

### Individual genes within the ORF of JEV strains exhibit differences in codon usage

To determine whether codon usage differed among JEV strains according to genotype and individual gene, the G+C content at the first and second codon positions (GC12), the G+C content at the synonymous third codon positions (GC3) and the codon usage index (*N*_C_) values were determined for all 32 virus strains over the ORF for all genotypes, as well as according to genotype and gene (Table 5). The mean GC12 value was 0.51 and ranged from 0.47 (GII|prM gene, GIII|prM gene and GIV|prM gene) to 0.54 (GIII|NS4B gene, GI|NS2B gene and GIV|NS2B gene). The GC3 values ranged from 0.46 (GII|NS2B gene) to 0.59 (GII|NS2A gene and GIV|NS2A gene), with a mean value of 0.52. The mean *N*_C_ value was 55.81 and ranged from 45.07 (GII|NS4A gene) to 61.00 (GI|NS2B gene, GIV|prM gene and GIV|NS4A). Therefore, the extent of codon usage bias was small, although some variation was present.

**Fig. 2.**
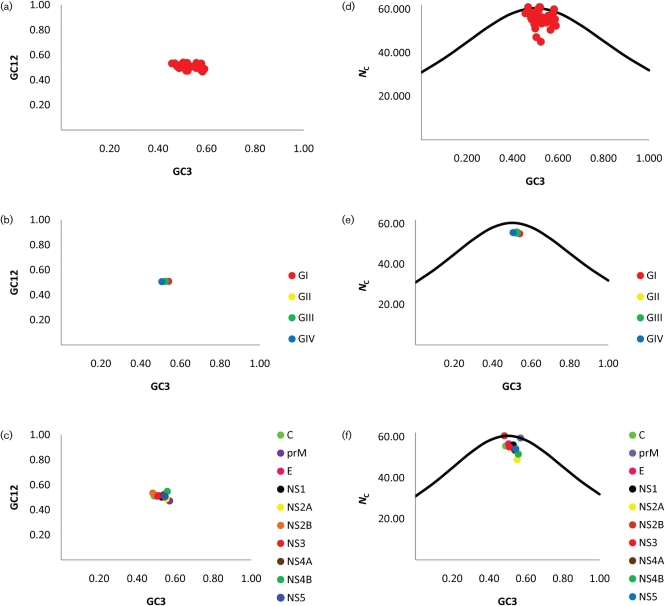
Correlation between GC12 and GC3 among all genotypes and genes (a), as well as according to genotype (b) and gene (c), and distribution of GC3 and *N*_C_ among all genotypes and genes (d), as well as according to genotype (e) and gene (f). The curves indicate the expected codon usage if G+C content constraints alone accounted for codon usage bias.

To determine whether the observed codon usage bias was controlled by mutational pressure or natural selection, the observed codon usage bias was compared with the bias that would be expected under the null hypothesis that mutation pressure is the sole determinant. First, GC3 was plotted against GC12 for all genotypes and genes (overall) (Fig. 2a), as well as according to genotype (Fig. 2b) and gene (Fig. 2c). A marginally statistically significant negative correlation between GC3 and GC12 was observed overall (*r*=−0.33, *P*=0.04), suggesting that patterns of base composition may be the result of mutational pressure. However, no statistically significant correlations between GC3 and GC12 were observed according to genotype (*r*=0.76, *P*=0.24) or gene (*r*=−0.43, *P*=0.22), indicating that patterns of base composition are most probably not the result of mutational pressure, as the effects are not present at all codon positions. Secondly, GC3 was plotted against *N*_C_ for all genotypes and genes (Fig. 2d), as well as according to genotype (Fig. 2e) and gene (Fig. 2f). For each plot, a curve was drawn representing the expected codon usage if G+C compositional constraints alone were responsible for the codon usage bias. In the plot of GC3 versus *N*_C_ over all genotypes and gene, the vast majority of points lay under the curve, whilst a few points lay on the curve and above the curve (Fig. 2d). When GC3 was plotted against *N*_C_ according to genotype, all points were positioned slightly below the curve, suggesting that the actual codon usage indices were close to the values expected from their G+C content and, furthermore, that no difference exists among the four genotypes of JEV (Fig. 2e). However, in the plot of GC3 versus *N*_C_ according to gene, some points overlapped the curve (NS2B and prM genes), whilst others lay further away (NS2A gene), suggesting that base composition as well as other factors may contribute to the observed codon usage bias (Fig. 2f).

## DISCUSSION

The known geographical range of JEV activity was historically limited to East and South-east Asia and Indonesia. Yet, in recent years, the range of virus activity has expanded west into Pakistan (Igarashi *et al.*, 1994) and south-east into Australia (Hanna *et al.*, 1996). In April of 1995, an outbreak of three human cases resulting in two fatalities occurred on Badu Island, which is located in the Torres Strait between mainland Queensland, Australia, and Papua New Guinea (Anon., 1995). Subsequent investigations revealed that the outbreak was caused by GII of the virus (Hanna *et al.*, 1996) and was probably introduced from Papua New Guinea by wind-blown mosquitoes or migratory birds (Johansen *et al.*, 2000). GII strains continued to be isolated from islands located in Australia's Torres Strait until 1998 (Pyke *et al.*, 2001), and a single GII strain was isolated in Malaysia in 1999 (CNS138-11, Malaysia, 1999) (Solomon *et al.*, 2003). However, in January 2000, a new genotype of JEV (GI) emerged in the Torres Strait (Pyke *et al.*, 2001). As GII of JEV has not been isolated since 1999, it remains unknown whether this genotype is still circulating or has become extinct. It is important to note that, following the collection of the prototype strain of GI of JEV in 1967 (M859/Cambodia/1967/Mosquito), another GI strain was not isolated until 1979, and this genotype has recently replaced GIII as the most frequently isolated genotype throughout a number of Asian countries (Nga *et al.*, 2004; Nitatpattana *et al.*, 2008). To understand better the genetic variation, phylogenetic relationships and evolution among strains of JEV, the nucleotide sequences of the ORF of three GII strains collected between 1951 and 1978 were determined and compared with sequence information derived from a strain collected during the 1995 JEV outbreak in Australia (FU, Australia, 1995), as well as with strains representing the other three genotypes of the virus.

It is noteworthy that West Nile virus (WNV), a closely related mosquito-borne flavivirus within the Japanese encephalitis serocomplex, also exists in endemic (Africa, Australia and Papua New Guinea) and epidemic (Europe) transmission cycles, as does JEV. GI and GIII of JEV typically occur in epidemic regions (i.e. north of southern Thailand), whilst GII and GIV occur mostly in endemic regions (i.e. south of southern Thailand) (Solomon *et al.*, 2003). The phylogeny presented here illustrates that, although GIII viruses have been introduced several times into the same locations (e.g. Taiwan), they are also maintained year to year, suggesting overwintering of the virus [e.g. Taiwan, 1958–1965 (HV1, TC and TL strains) and Taiwan, 1990–1997 (CH1392 and T1P1 strains)]. The phylogenetic tree also illustrated that all four of the Indian strains [GP78 (1978), 014178 (2001), 04940-4 (2002) and 57434 (2005)] grouped together within GIII, suggesting that JEV may have been introduced successfully into India only once and maintained there for at least 27 years. Furthermore, to the best of our knowledge, no other genotypes of the virus have ever been isolated from India. However, relatively few strains of JEV from India have been sequenced and this assertion may need to be revisited once additional nucleotide sequence data from this country become available. Interestingly, like JEV, WNV appears to have been introduced successfully into India only once (May *et al.*, 2011). It remains elusive why both JEV and WNV have been successfully introduced into India on only one occasion.

Nucleotide and amino acid pairwise comparisons over the ORF, as well as phylogenetic analyses, revealed that GII was the most similar to GI. Interestingly, GI has been isolated in East Asia, South-east Asia, Indonesia and northern Australia, whereas GII has been isolated on only two occasions in geographical areas where Japanese encephalitis is considered to be epidemic, i.e. Korea (Bennett strain, *c*.1951) and Australia (Badu island, FU strain, 1995). JEV utilizes a variety of vector species and hosts; geographical differences in vector or host availability could explain why the geographical range of GII of JEV is/was limited to tropical regions where JEV is endemic. Furthermore, it remains unclear whether GI has replaced GII in Australia and Papua New Guinea or whether both virus genotypes are circulating. To the best of our knowledge, no JEV isolates from these countries have been found or sequenced recently.

Correlation of the topology of the phylogeny with amino acid alignments of the JEV strains further illustrated the close evolutionary relationship between GI and GII of the virus; GI and GII shared 27 node-defining amino acid substitutions, whilst GII possessed 23 genotype-specific substitutions and GI had 21 genotype-specific substitutions. The low magnitude of genetic variation among the four geospatiotemporally distributed GII strains of JEV is consistent with the other three genotypes and indicates that the overall genomic tolerance for mutation is minimal. The NS2B protein was conserved among all GII strains. The central region of the NS2B protein of flaviviruses is a co-factor of the serine protease of the NS3 protein and is thus necessary for the activation of this protease (Mastrangelo *et al.*, 2007).

Selection analyses revealed that JEV strains are under predominantly purifying selection, which results in virus-encoded proteins being conserved over time due to selective pressure against deleterious variants. Evidence of strong positive selection was detected at aa 24 of the NS4B protein in the ORF nucleotide sequence file by three of the four detection methods and in the NS4B gene nucleotide sequence files by two of the four detection methods. This signifies that mutations at this amino acid over the evolutionary history of JEV have tended to be beneficial, rather than neutral or deleterious. The NS4B protein of flaviviruses has been found to inhibit the alpha/beta interferon (IFN-*α*/*β*) signalling cascade at the level of signal transducer and activator of transcription (STAT) phosphorylation, suggesting that amino acid mutations at aa 24 of this protein may be important in the antagonization of the host IFN response to the virus (Muñoz-Jordán *et al.*, 2003, 2005). Aa 22 and 24 of the WNV NS4B protein, which are analogous to JEV residues 22 and 24, respectively, have been shown to control IFN-*α*/*β* resistance in HeLa cells expressing subgenomic replicons lacking the structural genes, although no effect was shown on the expression of full-length infectious genomes (Evans & Seeger, 2007). Although potentially interesting, it should be noted that the amino acids at residues 22 and 24 of the NS4B protein are different for WNV and JEV, so extrapolation must be made with care.

The results of the overall codon usage analysis revealed that patterns of base composition may be the result of mutational pressure; however, when the data were parsed according to genotype and gene, we found that patterns of base composition were probably not the result of mutational pressure as the effects were not present at all codon positions. Also, we found little variance in the extent of codon usage bias among the genotypes of JEV; however, individual genes of the virus varied to a small degree in their extent of codon usage bias.

In conclusion, this study elucidated the genetic variation, phylogenetic relationships, operation of selection and patterns of codon usage among strains of JEV, including three newly sequenced strains of GII that were temporally isolated in geographically distinct regions. Compared with other flaviviruses such as yellow fever virus, the nucleotide and amino acid divergence among and within the four genotypes of the virus is relatively low (von Lindern *et al.*, 2006). However, the four genotypes of JEV exhibited distinct epidemiological histories and geographical ranges of virus activity. At this time, no phenotypic differences among the four genotypes of the virus have been described. Once phenotypic properties of the genotypes have been delineated, the genotype-specific amino acid substitutions defined in this study may correlate with these properties. Identification of genotype-defining molecular determinants and their associated phenotypic properties is vital to understanding the evolution and epidemiology of the virus, and may have an impact on future vaccine development strategies.

## METHODS

### Virus stains.

Three JEV strains were sequenced in this study: the Bennett strain was obtained from the Walter Reed Army Institute of Research, Silver Spring, MD, USA, and the WTP-70-22 (Malaysia, 1970) and JKT654 (Indonesia, 1978) strains were obtained from the World Reference Center of Emerging Viruses and Arboviruses at the University of Texas Medical Branch, Galveston, TX, USA (Table 1).

### Cell culture, virus growth, RNA extraction, RT-PCR, DNA purification and sequencing.

Viruses were inoculated onto confluent monolayers of *Aedes albopictus* C6/36 mosquito cells maintained at 28 °C in Dulbecco's modified essential medium containing 2 % FBS supplemented with 5 % tryptose phosphate buffer. Culture supernatant was harvested between 4 and 7 days post-inoculation. Viral RNA was extracted from the culture supernatant using a QIAamp Viral RNA Mini kit (Qiagen). The entire ORF (10 296 nt) of the viruses was amplified using the Titan One Tube RT-PCR System (Roche) and overlapping primer pairs (see Supplementary Table S3, available in JGV Online). We did not amplify the UTRs, as there is extensive variation within the 3′UTR of JEV strains, at least some of which seems to depend on the passage history of the viruses, whilst the 5′UTR is highly conserved among strains (Thurner *et al.*, 2004) and cannot be used for phylogenetic analyses. The PCR products were excised from an ethidium bromide-stained agarose gel and purified using a QIAquick Gel Extraction kit (Qiagen). Purified DNA was sequenced directly by standard methods at the Recombinant DNA Laboratory at the University of Texas Medical Branch at Galveston, TX, USA, using the PCR primers and sequencing primers (Supplementary Table S3).

### Phylogenetic and sequence analyses.

The nucleotide sequences of the three viruses were assembled and edited using ContigExpress (Vector NTI; Invitrogen) and deposited in GenBank. Newly generated JEV ORF sequences, as well as all available ORF sequences derived from wild-type, time-stamped strains retrieved from GenBank in September 2009 (29 strains) along with that of MVEV (strain MVE-1-51, used as an outgroup in the phylogenetic analyses) were aligned using AlignX (Vector NTI; Invitrogen) (Table 1). BioEdit (Hall, 1999) was used to adjust the nucleotide sequence alignment  manually to ensure that it was consistent with the ORF, to create individual nucleotide sequence alignments according to genotype and gene, to generate a deduced amino acid sequence alignment and to perform nucleotide and amino acid pairwise comparisons.

Phylogenetic relationships were inferred using NJ and ML methods in the phylip package (Felsenstein, 1989) and the Bayesian method in MrBayes v3.1.2 (Ronquist & Huelsenbeck, 2003). modeltest (Posada & Crandall, 1998) in conjunction with paup (Swofford, 1998) was used to identify the best-fit nucleotide substitution model that would be utilized in the phylogenetic analyses; the GTR+I+Γ_4_ model (general time-reversible+proportion of invariable sites+gamma-distributed rates in four categories, respectively) was chosen from a total of 56 models using three model selection frameworks (hierarchical likelihood ratio tests, Akaike information criterion and Bayesian information criterion). The NJ analysis employed the nucleotide substitution model and F84 distance matrix algorithm, whilst the ML analysis utilized the GTR+I+Γ_4_ nucleotide substitution model in conjunction with a heuristic search algorithm; the robustness of the NJ phylogeny was evaluated by bootstrap resampling with 1000 replicates, whilst the robustness of the ML phylogeny was evaluated by bootstrap resampling with 100 replicates. The Bayesian method used the GTR+I+Γ_4_ nucleotide substitution model; the nucleotide data were partitioned by first, second and third codon positions separately and the substitution parameters were allowed to vary across partitions. The analysis used three hot chains and one cold chain, and ran twice for 1.6 million generations with 10 % burn-in; the ‘sump’ command and Tracer v1.4.1 (Rambaut & Drummond, 2005) were used to ensure model convergence. The robustness of the Bayesian phylogeny was evaluated by determining posterior probabilities for each of the nodes within the tree.

### Recombination and selection analyses.

The presence of recombination in a nucleotide sequence alignment can confound attempts to estimate selection pressures; therefore, recombination analyses were performed as a prerequisite to the selection analyses. Recombination among the sequences was analysed using the rdp (Martin & Rybicki, 2000), geneconv (Padidam *et al.*, 1999), Chimaera (Posada & Crandall, 2001), MaxChi (Smith, 1992) and Bootscan (Martin *et al.*, 2005a) methods implemented in the rdp3 v*β*41 program (Martin *et al.*, 2005b). Common program settings for all methods were to perceive sequences as linear, to require phylogenetic evidence, to refine breakpoints and to check alignment consistency. The highest acceptable *P* value was set at 0.05, after considering Bonferroni correction for multiple comparisons. All method-specific program settings remained at their default values. Only recombination events that were identified by at least two methods were considered as potential recombination events. The breakpoint positions and recombinant sequence inferred for every detected potential recombination event were verified manually using phylogenetic and recombination signal analysis features in rdp3.

Evidence of non-neutral selection was examined by calculating the *d*_N_/*d*_S_ ratio using a NJ phylogeny and the general reversible nucleotide substitution model available through the Datamonkey web server (Pond & Frost, 2005). Four methods were used to detect non-neutral selection: single-likelihood ancestor counting (SLAC), FEL, REL and IFEL (Kosakovsky Pond & Frost, 2005). A set of 44 nt sequence alignment files that were parsed according to protein and genotype were used in the analyses.

### Codon usage analysis.

The analysis of codon usage patterns was performed on a set of 44 nt sequence alignment files, which were created by dividing the ORF dataset according to genotype and gene, using the CodonW software package (http://codonw.sourceforge.net/).

GC12, GC3 and *N*_C_ were calculated for each virus in all of the datasets and the means determined. The reported value of *N*_C_ is always between 20 (when only one codon is used for each amino acid) and 61 (when all codons are used equally). The relationship between GC3 and GC12 was examined to determine the relative effects of mutation pressure versus natural selection on codon composition. Pearson's *r* correlation coefficient was used to measure the linear relationship between the two interval variables, GC12 and GC3, by employing the pasw Statistics 18 software program. To examine the influence of G+C content on codon usage, the relationship of *N*_C_ and GC3 was plotted. This was compared with the *N*_C_ value that would result if G+C content were solely responsible for the codon biases, calculated as *N*_C_=2+GC3+(29/[(GC3)^2^+(1−GC3)^2^]) (Wright & Bibb, 1992).

## Supplementary Material

[Supplementary Tables]

## Figures and Tables

**Table 1.** Details of the virus strains used in this study

**Strain**	**Geographical origin**	**Year**	**Host**	**Genotype**	**GenBank accession no.**
K94P05	Wando Island, Chullaman-Do, Korea	1994	Mosquito	GI	AF045551
Ishikawa	Ishikawa, Japan	1998	Mosquito	GI	AB051292
KV1899	Gyeonggi, Korea	1999	Swine	GI	AY316157
JEV/sw/Mie/41/2002	Mie, Japan	2002	Swine	GI	AB241119
JEV/sw/Mie/40/2004	Mie, Japan	2004	Swine	GI	AB241118
XJP613	China	2007	Mosquito	GI	EU693899
HEN0701	China	2007	Swine	GI	FJ495189
SH17M-07	China	2007	Unknown	GI	EU429297
XJ69	ZheJiang, China	2007	Mosquito	GI	EU880214
JKT654*	Java, Kapuk, Indonesia	1978	Mosquito	GII	HQ223287
Bennett*	Korea	*c*.1951	Human	GII	HQ223285
WTP-70-22*	Kuala Lumpur, Malaysia	1970	Mosquito	GII	HQ223286
FU	Badu Island, Australia	1995	Human	GII	AF217620
Nakayama	Nakayama, Japan	1935	Human	GIII	EF571853
Beijing-1	Beijing, China	1949	Mosquito	GIII	L48961
SA14	Xian, China	1954	Mosquito	GIII	U14163
HV1	Taiwan	1958	Human	GIII	AF098735
JaGAr01	Gunma, Japan	1959	Mosquito	GIII	AF069076
JaTH160	Tokyo, Japan	1960	Human	GIII	AB269326
Ling	Taiwan	1965	Human	GIII	L78128
TC	Taiwan	1965	Human	GIII	AF098736
TL	Taiwan	1965	Human	GIII	AF098737
JaOH0566	Osaka, Japan	1966	Human	GIII	AY508813
GP78	Gorakhpur, India	1978	Human	GIII	AF075723
JaOArS982	Osaka, Japan	1982	Mosquito	GIII	M18370
K87P39	Wando Island, Chullaman-Do, Korea	1987	Mosquito	GIII	U34927
CH1392	Changhua City, Taiwan	1990	Mosquito	GIII	AF254452
T1P1	Liu-Chiu Islet, Taiwan	1997	Mosquito	GIII	AF254453
014178	Lakhimpur, India	2001	Human	GIII	EF623987
04940-4	Maharashtra, India	2002	Mosquito	GIII	EF623989
057434	Gorakhpur, India	2005	Human	GIII	EF623988
JKT6468	Flores, Golock, Indonesia	1981	Mosquito	GIV	AY184212
MVE-1-51†	Australia	1951	Human	–	AF161266

*Isolates sequenced in this study. †All strains are JEV except for MVE-1-51, which is a strain of MVEV.

**Table 2.** Nucleotide and amino acid sequence divergence among the JEV strains

**Genotype**	Divergence (%) from:*						
	**GI (nine strains)**	**GII (Bennett)**	**GII (WTP-70-22)**	**GII (JKT654)**	**GII (FU)**	**GIII (nine strains)**	**GIV (JKT6468)**
GI (nine strains)		**1.5–2.9**	**1.7–3.0**	**1.7–3.1**	**1.9–3.3**	**1.6–3.8**	**5.0–6.2**
GII (Bennett)	9.1–9.7		**0.5**	**0.5**	**0.8**	**1.7–2.6**	**5.1**
GII (WTP-70-22)	9.5–10.1	2.5		**0.5**	**0.8**	**1.9–2.8**	**5.0**
GII (JKT654)	9.8–10.4	3.1	2.8		**0.9**	**1.9–2.8**	**5.1**
GII (FU)	10.1–10.7	4.0	3.3	4.3		**2.0–3.0**	**5.4**
GIII (18 strains)	10.8–11.9	10.2–10.9	10.5–11.3	10.7–11.5	10.9–11.7		**4.6–5.5**
GIV (JKT6468)	16.6–17.2	16.2	16.4	16.6	16.6	15.4–16.1	

*Nucleotide divergence is in normal type and amino acid divergence is in bold type.

**Table 3.** Amino acid substitutions within the ORF of GII strains of JEV

**Protein**	**Aa residues relative to ORF**	**Length of protein**	**Aa substitution relative to protein**	**Strain**			
				**Bennett**	**WTP-70-22**	**JKT654**	**FU**
C	1–127	127	44	V	A	A	A
			90	T	T	T	I
			123	A	V	G	A
prM	128–294	167	58	P	S	S	T
E	295–794	500	108	F	F	F	S
			126	I	T	I	I
			208	S	S	S	P
			307	K	K	K	N
			308	F	F	F	S
			311	A	A	A	R
			366	S	S	S	A
NS1	795–1146	352	50	H	Y	Y	Y
			79	L	F	L	L
			214	R	R	K	R
			240	E	E	E	D
			284	K	K	K	T
			326	E	E	E	D
			338	V	V	V	A
NS2A	1147–1373	227	61	T	A	A	A
			134	I	I	T	I
			188	K	K	R	K
			220	I	V	V	A
NS2B	1374–1504	131					
NS3	1505–2123	619	62	E	G	G	G
			117	R	C	R	R
NS4A	2124–2272	149	96	A	A	T	A
NS4B	2273–2527	255	8	K	K	K	R
			31	S	I	S	S
			74	S	S	A	S
NS5	2528–3432	905	3	P	P	A	P
			135	K	R	R	K
			155	E	E	E	D
			298	S	S	S	P
			398	R	K	K	K
			429	G	S	G	D
			5887	A	A	A	S
			682	A	A	A	S
			835	I	V	I	I
			878	V	I	V	I
			897	A	V	A	V

**Table 4.** Evidence for positive and negative selection using four detection methods

**Protein**	**Aa residues relative to ORF**	**Length of protein**	**Genotype**	**Overall *d*_N_/*d*_S_**	SLAC*		FEL*		**REL†**		IFEL*	
					**Positive selection**	**Negative selection**	**Positive selection**	**Negative selection**	**Positive selection**	**Negative selection**	**Positive selection**	**Negative selection**
ORF	1–3432	3432	GI–IV	0.035	0	807	1	1432	10	1204	1	855
			GI	0.067	0	19	0	143	0	0	0	13
			GII	0.020	0	12	0	232	0	43	0	0
			GIII	0.057	0	126	0	383	1	29	0	112
C	1–127	127	GI–IV	0.147	0	14	0	26	0	54	0	16
			GI	0.111	0	0	0	5	0	13	0	0
			GII	0.133	0	0	0	4	3	0	0	0
			GIII	0.166	0	1	0	5	0	0	0	2
prM	128–294	167	GI–IV	0.048	0	33	0	78	0	0	0	41
			GI	0.061	0	0	0	8	1	0	0	0
			GII	0.015	0	0	0	20	1	35	0	0
			GIII	0.083	0	6	0	19	0	0	0	6
E	295–794	500	GI–IV	0.033	0	135	0	225	0	0	0	141
			GI	0.069	0	4	0	23	0	77	0	4
			GII	0.023	0	3	0	42	0	0	0	0
			GIII	0.065	0	21	0	58	0	129	0	19
NS1	795–1146	352	GI–IV	0.043	0	81	0	145	0	0	1	83
			GI	0.041	0	13	0	49	0	24	0	10
			GII	0.031	0	3	0	28	0	55	0	0
			GIII	0.061	0	14	0	52	0	6	0	12
NS2A	1147–1373	227	GI–IV	0.052	0	46	0	81	0	0	0	46
			GI	0.047	0	3	0	13	0	24	0	2
			GII	0.050	0	1	0	11	1	1	0	0
			GIII	0.069	0	6	0	19	0	55	0	6
NS2B	1374–1504	131	GI–IV	0.014	0	31	0	65	0	0	0	32
			GI	0.012	0	0	0	5	0	0	0	0
			GII	5.310×10^−16^	0	0	0	7	0	0	0	0
			GIII	0.038	0	6	0	14	0	0	0	4
NS3	1505–2123	619	GI–IV	0.025	0	167	0	285	0	3	0	186
			GI	0.058	0	2	0	25	0	0	0	0
			GII	0.006	0	0	0	46	0	0	0	0
			GIII	0.054	0	25	0	72	0	172	0	29
NS4A	2124–2272	149	GI–IV	0.045	0	31	0	57	1	90	0	23
			GI	0.080	0	0	0	7	0	0	0	1
			GII	0.012	0	0	0	8	0	0	0	0
			GIII	0.073	0	4	0	14	3	16	0	4
NS4B	2273–2527	255	GI–IV	0.025	0	61	1	130	0	0	1	64
			GI	0.056	0	1	0	11	0	0	0	2
			GII	0.019	0	2	0	14	0	0	0	0
			GIII	0.035	0	9	0	37	2	21	0	8
NS5	2528–3432	905	GI–IV	0.033	0	210	0	373	1	47	0	243
			GI	0.081	0	3	0	35	0	109	0	1
			GII	0.025	0	1	0	46	0	144	1	0
			GIII	0.014	0	5	0	21	0	48	0	5

*Number of sites where *P*<0.05. †Number of sites where Bayes factor >100.

**Table 5.** GC12, GC3 and *N*_C_ values according to genotype and gene

**Gene**	**All**			**GI**			**GII**			**GIII**			**GIV**		
	**GC12**	**GC3**	***N*_C_**	**GC12**	**GC3**	***N*_C_**	**GC12**	**GC3**	***N*_C_**	**GC12**	**GC3**	***N*_C_**	**GC12**	**GC3**	***N*_C_**
ORF	0.51	0.53	55.55	0.51	0.54	55.13	0.51	0.52	55.85	0.51	0.53	55.68	0.51	0.51	55.73
C	0.51	0.49	55.52	0.51	0.51	47.23	0.52	0.51	60.06	0.51	0.48	58.79	0.50	0.50	53.19
prM	0.47	0.57	59.45	0.48	0.58	57.98	0.47	0.52	59.73	0.47	0.58	60.03	0.47	0.52	61.00
E	0.51	0.51	56.56	0.51	0.48	57.26	0.51	0.48	55.82	0.51	0.53	56.40	0.50	0.49	56.11
NS1	0.50	0.52	56.08	0.50	0.53	56.44	0.50	0.51	56.71	0.50	0.52	55.82	0.50	0.49	55.30
NS2A	0.50	0.57	54.18	0.50	0.58	53.47	0.49	0.59	52.44	0.50	0.56	54.81	0.49	0.59	55.43
NS2B	0.53	0.48	60.54	0.54	0.52	61.00	0.53	0.46	58.16	0.53	0.47	60.98	0.54	0.50	58.11
NS3	0.51	0.51	55.04	0.51	0.53	53.44	0.51	0.51	54.86	0.51	0.51	55.84	0.52	0.49	55.75
NS4A	0.52	0.53	53.80	0.52	0.57	50.58	0.51	0.53	45.07	0.52	0.51	56.78	0.52	0.52	61.00
NS4B	0.54	0.55	56.20	0.53	0.58	54.89	0.53	0.51	55.85	0.54	0.56	57.13	0.53	0.50	51.22
NS5	0.51	0.55	54.17	0.51	0.56	54.31	0.51	0.55	53.86	0.51	0.54	54.09	0.50	0.53	55.50
