# Supplementary material for: Genetic characterization of Japanese encephalitis virus genotype II strains isolated from 1951 to 1978
Source: J Gen Virol. 2011 Mar;92(Pt 3):516–27. doi: 10.1099/vir.0.027110-0 (PMC3081233; doi:10.1099/vir.0.027110-0)
Supplement: [Supplementary Tables] [file supp_92_3_516__index.html]

 Genetic characterization of Japanese encephalitis virus genotype II strains isolated from 1951 to 1978 -- Schuh et al. 92 (3): 516 Data Supplement - Supplementary Tables -- Journal of General Virology

## 

### Genetic characterization of Japanese encephalitis virus genotype II strains isolated from 1951 to 1978, by A. J. Schuh, R. B. Tesh and A. D. T. Barrett

*Journal of General Virology* vol. **92**, part 3, pp. 516 - 527

**Supplementary Table S1.** Genotype-specific and node-defining amino acids within the ORF of JEV strains

**Supplementary Table S2.** Confirmed recombination events

**Supplementary Table S3.** Primers used to amplify and sequence the ORF of the JEV strains   
  
 [Single PDF file]  (115 KB)

  
  
