# Supplementary material for: Genetic characterization of Japanese encephalitis virus genotype II strains isolated from 1951 to 1978
Source: J Gen Virol. 2011 Mar;92(Pt 3):516–27. doi: 10.1099/vir.0.027110-0 (PMC3081233; doi:10.1099/vir.0.027110-0)
Supplement: [Supplementary Tables] [file supp_92_3_516__1.pdf]

Supplementary Table S1. Genotype-specific and node-defining amino acids within the ORF of JEV strains

Protein	aa residues relative to the ORF	Length of protein	aa residue relative to protein	Consensus	Genotype			
					GI	GII	GIII	GIV
C	1–127	127	6	G				R
			10	C				I
			15	N				Y
			35	M				I
			41	R				I
			44	V				L
			51	I				V
			52	T				S
			60	A				S
			70	R			K	
			72	V				L
			100	R	K	K		
			109	E				G
			110	G	S	S		
			111	S				T
			112	I				T
			113	M				L
			115	L				F
			116	A				M
			120	V	I	I	V	I
			121	V				A
			122	I	T	M	I	A
			123	A				V
			125	A				V
prM	128–294	167	1	M				L
			13	T				A
			14	I				V
			57	T	A			
			58	M	V			P
			63	E				Q
			71	N				H
			76	V		I		
			85	R				S
			88	K				Q
			89	R				T
			97	Q				H
			113	D				N
			122	M				V
			129	I				V
			140	V	A	A		
			149	N	S			S
			151	Q				P
E	295–794	500	15	A				V
			38	K				R
			126	I				T
			128	R				K
			129	T	M			
			156	S				T

			159	V				I
			171	V				I
			222	A	S	S		
			228	P				S
			230	S				N
			260	G				R
			261	G				A
			327	S	T	T	S	L
			366	A	S	S/A (FU)	A	S
			369	K				Q
			389	D				E
			399	A				P
			466	A				V
			473	V				I
			482	L				M
			486	A				V
			490	V				T
			492	V				L
NS1	795–1146	352	41	S				A
			50	H		Y/H (Bennett)		
			51	K	Q/K (KV1899)			M
			54	V				I
			57	V		I		
			70	A	S	S		
			147	H	R/H (KV1899)	R	H	H
			175	S	N/T (KV1899)	S	S	N
			182	A				T
			188	V				I
			205	R				H
			206	Y	L/Y (KV1899)	F	Y	L
			220	V				I
			242	I		V		
			251	K	R	R		
			271	N				D
			298	V	I/L (KV1899)			
			317	S				T
			326	E				G
			327	N				S
			335	I				V
			339	R				K
			343	T				A
			350	V				A
NS2A	1147–1373	227	2	N				S
			6	V	I/V (KV1899)	I	V	I
			34	I		V		V
			70	S	S	N	S/N (K87P39)	N
			92	M				A
			97	T	A	A		
			98	R		K		

			105	V		M		
			119	V				A
			128	I				M
			139	V				I
			140	R				K
			149	S	T	T	S	T
			150	V				I
			151	T	A			
			154	V				L
			159	T				A
			176	V				I
			179	I				V
			187	K	R	R	K/E (HV1)	R
			188	K				R
NS2B	1374–1504	131	55	E	D			
			65	D	E			
			97	V		I		
			99	V	L	L		
NS3	1505–2123	619	14	S	L/S (KV1899)	L	S/L (K87P39)	A
			44	N				S
			62	E		G/E (Bennett)		
			78	A	S	S		
			105	A	P			
			107	V				T
			117	R				C
			175	V				I
			177	E	D			
			180	T				N
			182	N	S	S	N	S
			185	F	K	K	F	K
			210	K				R
			294	S				G
			340	I				V
			354	E	D			
			356	A		S		
			374	M				V
			495	M				L
			586	M				T
			591	I				V
NS4A	2124–2272	149	3	V				I
			17	M				A
			35	K				R
			58	V		I		
			61	T				A
			72	M				K
			88	T				V
			100	P				S
			110	I	V/I (K94P05)			
NS4B	2273–2527	255	20	K	R/K (K94P05)			
			21	T				A
			22	Q				P

Schuh, A. J., Tesh, R. B. and Barrett, A. D. T. (2011). Genetic characterization of Japanese encephalitis virus genotype II strains isolated from 1951 to 1978. *J Gen Virol* **92**, 516–527.

			23	A				V
			24	S	P (five strains)/S (four strains)	P		
			26	L				M
			31	S				G
			59	L				I
			73	N	S	S		
			115	M				V
			118	V	V	V	A (15 strains)/V (three strains)	T
NS5	2528–3432	905	15	K				R
			22	E	D	D		
			49	I				K
			78	I				V
			101	R	K/R (K94P05)	K		
			135	K				R
			144	F				L
			253	K		R		
			275	N		D		D
			277	E				G
			280	K	R			
			287	K	R/K (three strains)			
			296	K	R			
			298	P		S/P (Bennett)		
			370	A				P
			372	A	V	V	A	V
			381	H				Y
			429	D	G/S (two strains)/D (one strain)	G/S (one strain)/D (one strain)	D/N (two strains)	G
			432	R	L/R (K94P05)			
			438	D	N/D (K94P05)			
			453	I		V		V
			455	N				H
			503	E				R
			526	G				R
			528	Q				E
			546	R				K
			576	R				K
			586	A				S
			587	A				T
			588	E	G	G	E	D
			637	L				V
			644	N		T		
			661	T				S
			684	H				Y
			754	K				R

	787	A				T
	830	M		T		E
	860	S				R
	878	V	I/V (SH17M-O7)	I (two strains)/M (two strains)		
	883	N				T
	889	T		L		

Supplementary Table S2. Confirmed recombination events

NS, Not significant at *P*>0.05.

Recombination event	Gene	Nt breakpoint positions in recombinant	Recombinant	Minor parent	Major parent	Algorithm	Recombination *P* value
1	NS5	8838–9063	K94P05	JaOArS982	JEV/sw/Mie/40/2004	RDP	2.63×10^{-27}
						GENECONV	8.27×10^{-26}
						Bootscan	1.20×10^{-24}
						Maxchi	2.40×10^{-8}
						Chimaera	9.23×10^{-5}
2	NS5	7883–8072	K94P05	JaOArS982	XJ69	RDP	4.68×10^{-19}
						GENECONV	4.28×10^{-18}
						Bootscan	3.75×10^{-19}
						Maxchi	9.44×10^{-5}

						Chimaera	9.53×10^{-5}
3	NS4A-NS4B	6697–6880	K94P05	JaOArS982	SH17M-07	RDP	2.05×10^{-12}
						GENECONV	4.25×10^{-12}
						Bootscan	1.73×10^{-12}
						Maxchi	0.02
						Chimaera	1.05×10^{-2}
4	NS3	5903–6009	K94P05	JaOArS982	JEV/sw/Mie/41/2002	RDP	8.83×10^{-10}
						GENECONV	9.28×10^{-9}
						Bootscan	7.34×10^{-10}
						Maxchi	NS
						Chimaera	NS

Supplementary Table S3. Primers used to amplify and sequence the ORF of the JEV strains

**Primer name**	**Sequence (5′→3′)**
11S	CTGTGTGAACTTCTTGGCTT
484A	GTCCGTATTGTTGACAGCCA
94S	CCATGACTAAAAAACCAGGAGGGCC
996A	CCATTCCCAGGCAATTGAAGCTGTAAGC
F879	GCTTTCCTGGCGGCGGTACTTG
R2570	CCTATCCACCCAGGCTTCCACGTCG
F1468	CGGCAAAGTTTACAGTAACACCCAATGC

R2088	GCACCTTTGAGTTGGCACTGGAAGTC
842S	GCTTTCCTGGCGGCGGTACTTG
1192A	GCCACCGTCGAGATGTCAGTG
940S	TTACTATCCTTCTGCTGTTGGTCGCTCCG
1720A	GTGGCGTGCGCCTCTTCAAA
1271S	GACACATGTGCTAAGTTCTC
1639A	TCTATTTCTCCATGCTGTGT
1598S	CTCGAAGTCATTCTTAGTCCACAGG
2171A	TTGTGCCAGTGATGATTGATCTGC
2115S	GATTCTTACATCGTGGTCGGAAGACG
2624A	CGCTTTGTGGACAATCTTTGCTAAGG
2091S	GAGGAGAAAAACAAATCAACC
2518A	CCACACCTCATCTCTTTTCTTG
2534S	CTTCGTACACAACGATGTGGAAGCTTGGG
3200A	AAGATCACTTTCCTCAACGCCATCTCCC
3053S	GGCGGTCCATAGTGACTTGTCG
3591A	GGCCAGAAACATCACCAGAAGG
3172S	GGGGAGATGGCGTTGAGGAAAGTGATC
3866A	TGCCCCTAGGACCAAAACCATGTTTTCT
3301S	TGGACTTTGATTATTGCCCA
4131A	CCAAGAGTACAGCTCCTTTC
3837S	CAAGAAAACATGGTTTTGGTCCTAGGGGC
4458A	CATCATCCAGCTTAACATCTAGCCTCCGG
3976S	TCCACCATCACCATGCCATT
4499A	CATGGAACACCGGGATCATC
4421S	GGGAAGCAGCCGGAGGCTAGATGTTAA
5122A	TCTTGACGGTCACCTTGCACAATAGCG

5072S	Unknown
5745A	CTGCTCTCTGGAGGCACATTGCTATCT
5603S	AATCCACGATTTGCAGGATGAGATCCCAG
5964A	CACTGGTTATGGGAGACGGATTTCCAAGA
5857S	AATTTTGGAGCGAGCAGGGT
6407A	CTTGCATCAAGCCATCTGGG
6352S	TAGTCACCCGGATGGGTGAGAGAAAGATC
6742A	AAAAAGGTGGCCAGCGTGAGCACTAAA
6619S	AGACCATCACACTCATCATTGC
7212A	CAAGAAAGACAAGGCCAACG
6652S	TGACAGGAGGATTCTTCCTGCTCATGATG
7376A	AACCATTCCGTCTACGACGGCATTCTT
7097S	GGAATACGTCACCACATCATTAGC
7789A	GTGCGGTCCACCTCAATTATGG
7561S	TCTGGAATTCCACCACTGCCACAGGGC
8403A	GCCCTAACAGCACCTGGCTAGTCATGTTG
8194S	ACCGGGGACCTAGAGAGTTCTGC
8809A	TCGTTGAGCACTTCCTTGACTCC
8372S	CGGTGAACATGACCAGCCAG
9011A	AGGTGGTTTTCCCTCTCCAC
8714S	TGACCGACACAACCCCTTTTGG
9354A	CCAAAAGCTCCAGCACCTTGGC
9252S	AGGAAAGCAAGGAGGGAAAA
9588A	CACATGTTGTGGTCCAATAA
FU2	GCTGATGACACCGCCGGCTGGGACAC
CFD3	AGCATGTCTTCCGTGGTCATCCA
9751S	TCAGAAAAGACATCCAGGA

10347A	GTGAAGTCATGTAATCGACA
EMF1	TGGATGACSACKGARGAYATG
VD8	GGGTCTCCTCTAACCTCTAG
10201S	CATACGTGGGAAAGCGTGAGGACATCTGG
10944A	CCACCAGCTACATGTTTCGGCGCTC
